# The supportive supervisory scale: psychometric properties in Chinese health care aides samples

**DOI:** 10.1186/s12955-021-01706-y

**Published:** 2021-02-23

**Authors:** Li Tian, Haixia Li, Bei Dong, Congyan Xie, Hong Wang, Lu Lin

**Affiliations:** 1 The First Affiliated Hospital of Soochow University, Suzhou, 215006 People’s Republic of China; 2 School of Nursing, Medical College of Soochow University, No. 1 Shizi Street, 215006 Suzhou, People’s Republic of China; 3 Suzhou Jiulong Hospital, Shanghai Jiao Tong University School of Medicine, Suzhou, People’s Republic of China

**Keywords:** Supportive supervisory scale, Chinese version, Psychometric properties, Health care aides

## Abstract

**Objective:**

To sinicize the Supportive Supervisory Scale (SSS) and analyze the psychometric properties of the Chinese version of SSS (SSS-C).

**Methods:**

The SSS (the original English version) was firstly sinicized and adjusted, then its psychometric properties were examined in 300 health care aides from four long-term care (LTC) facilities. SPSS 22.0 was used to process the data and calculate the reliability and validity.

**Results:**

The 15-item SSS-C had satisfactory internal consistency (Cronbach’s α coefficient = 0.852), split half reliability (Spearman-Brown coefficient = 0.834) and test–retest reliability (Pearson correlation coefficient = 0.784), and three factors were extracted. If the four items with their communality < 0.4 were deleted, the remaining 11 items could explain 55.654% of the total variance. The discriminant validity of the SSS-C varied significantly between sites.

**Conclusions:**

The Chinese version of SSS can be used to effectively measure the supervisory support of the nurses within the LTC settings.

## Introduction

With the aging of global population, the needs for long-term care (LTC) have increased significantly. However, most of long-term care facilities (LTCFs) have been faced with the staffing challenges, resulting in poor capacity to provide competent and high-quality long-term care to the elderly [1–3]. Health care aides (HCAs, equivalent to nursing assistants) provided 80–90% of the direct care to LTCF residents [4]. Thus, it can be seen that the stability and quality of HCAs affect the nursing care of LTCFs to a considerable extent. In LTCFs, HCAs are often supervised by registered nurses, and evidence is accumulating that these supervisory relationships prominently affect HCA turnover, job satisfaction, and the quality of the care provided [3, 5, 6]. In addition, supportive supervisory practices have been proven to be associated with patient outcomes, for example, less adverse events and complications [7]. Therefore, it is urgent to measure and improve the supervisory support of HCA supervisors in the LTCFs.

Supportive Supervisory Scale (SSS) has been originally developed in English for this purpose and has been proven to be a reliable, valid, and useful tool to assess the supervisory support of supervisors in LTCFs, which may influence the retention of HCAs and quality of resident care [8]. To date, such instruments to evaluate the supervisory support of the supervisors within LTCFs are still lacking in China. Given the above needs, the authors obtained the authorization from the author of the original SSS scale, Prof. McGilton, and sinicized and adapted it, and then examined the psychometric properties of the Chinese version of SSS for use in China.

## Methods

This study was approved by the Ethics Committee of Soochow University (No. SUDA 20200515H03). All participants were given both verbal and written information about the study; those who agreed to participate in this study signed an informed consent.

### Instrument and sinicization

The SSS has 15 items and includes two parts. The first part is labeled “Respect Uniqueness” and the second part “Being Reliable” [9]. Answer options are “never”, “seldom”, “occasionally”, “often”, “always”, which successively represent the score of “1, 2, 3, 4, 5”.

**Fig.1:**
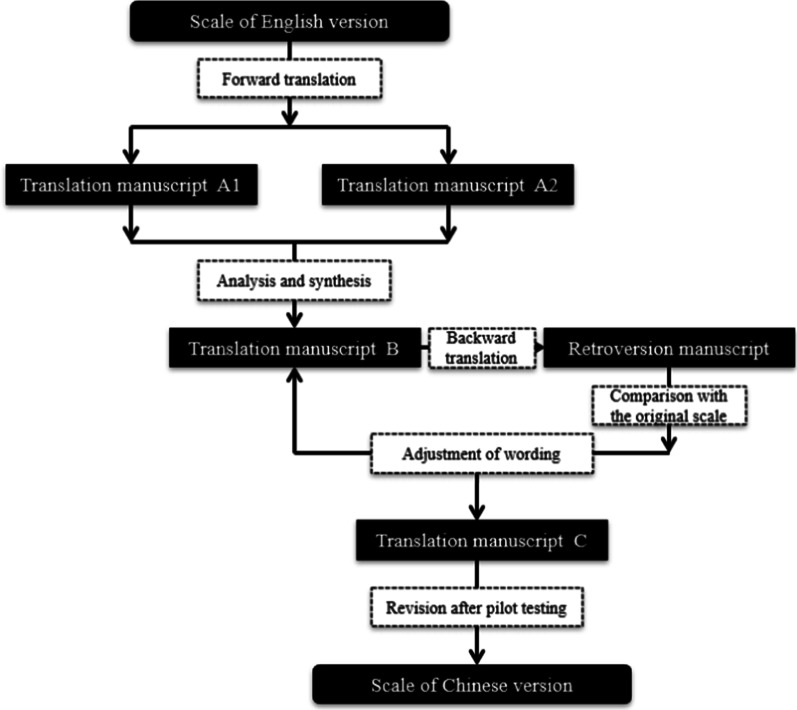
Sinicization steps of SSS

The SSS was translated from English into Chinese using Brislin’s translation model [10]. The steps for sinicization of SSS are shown in Fig. 1. Firstly, two bilingual researchers separately translated the original SSS into Chinese. The discrepancies between these two translations were reviewed and discussed comprehensively, and formed a single version, which was then translated back into English by another bilingual researcher. The retroversion was repeatedly compared with the original SSS scale and the Chinese expressions were adjusted accordingly. Throughout the Chinese version, “supervisor” was replaced by “nurse supervisor” to make the items more understandable since HCAs report directly to registered nurses in LTCFs in China. During this procedure, the translation validity index (TVI) was used to assess the translation equivalence of versions. It used a 4-point Likert scale (1 = uncorrected, 2 = needs major modification on equivalent item, 3 = equivalent but needs minor modification, and 4 = equivalent). In this study, three language experts were recruited to compare the SSS in English and Chinese. The items were revised until a TVI score of 4 was achieved. The revised version of SSS was pilot tested with a convenience sample of 30 HCAs in a LTCF in Suzhou to evaluate whether the Chinese version of SSS was easy to understand. Language expression was adjusted if HCAs felt it was difficult to understand. After the pilot test, the Chinese version of SSS (SSS-C) was finalized for the test of its psychometric properties.

### Sample and setting

The study was conducted in 4 LTCFs in Suzhou, China. Health care aides meeting the following criteria were enrolled in the study: (i) working in the LTCFs for more than 3 months; (ii) able to give written consent. A total of 300 participants completed the scale. 41.4% of the HCAs were less than 50 years old and 54% were 51–60 years old; 85.3% were female; 100% of the respondents were employed full-time. The mean number of years that the respondents have worked in the LTCF was less than 5 years (n = 241, 80.3%).

### Procedures

After giving written consent, HCAs were asked to fill out the survey questionnaire independently and anonymously in the nursing station or staff lounge, without their supervisor being present. It was guaranteed that the supervisors had no access to the responses. The researchers remained in the room and were available to answer questions when necessary. Neither compensation nor remuneration was offered to the participants.

### Statistical analyses

Analyses were performed by IBM SPSS Statistics 21. Participant characteristics and major variables were summarized by descriptive statistics (Table 1). Reliability was tested by internal consistency (Cronbach’s alpha), split-half reliability (Spearman-Brown coefficient) and test–retest reliability (Pearson correlation coefficient) (reliability coefficient ≥ 0.7 was acceptable) [11]. Construct validity was examined by exploratory factor analysis (EFA) (principal component analysis with varimax rotation). Scree plot, Kaiser criterion (eigenvalue ≥ 1.0), and clinical interpretability were considered in determination of factor solution (When the factor loading ≥ 0.40, the item can be considered in the factor) [12]. Discriminative validity was assessed by examining if supervisory support varied between the different facilities by one-way analysis of variance. Multiple comparisons, using Bonferroni’s procedure, were completed to compare every pair of facilities. The significant level was 0.05[13].

**Table 1:** Participant characteristics (*N* = 300)

Socio-demographic characteristics	Number (%)	Socio-demographic characteristics	Number (%)
Age		Original occupation	
≤ 30	3 (1)	Relevant occupation	104 (34.7)
31–40	8 (2.7)	Unrelated occupation	196 (65.3)
41–50	113 (37.7)	How the participant obtained this job	
51–60	162 (54.0)	Help from relatives or friends	159 (53)
≥ 61	14 (4.7)	Help from domestic companies	44 (14.7)
Gender		Recommended by employment center or relevant departments	20 (6.7)
Male	44 (14.7)	Official recruitment	111 (37.0)
Female	256 (85.3)	Others	18 (6.0)
Marital status		The reasons for doing this job	
Married (including remarriage)	281 (93.7)	No better work	46 (15.3)
Not married (single, divorced, and widowed)	19 (6.3)	Stable income	107 (35.7)
Education		Want to work in the city	23 (7.7)
Primary school	111 (37.0)	Happy to serve the elderly	148 (49.3)
Junior high school	149 (49.7)	Learning knowledge and skills	85 (28.3)
Senior high school and technical secondary school	36 (12.0)	Gain respect and praise	21 (7.0)
≥ Junior college	4 (1.3)	Others	7 (2.3)
Residence		Professional attitude	
Urban	30 (10.0)	Respectable	192 (64.0)
Town	82 (27.3)	No difference from other jobs	90 (30.0)
Rural	188 (62.7)	Low social status	18 (6.0)
Average monthly income		Change profession when possible	
< 3000 RMB	2 (0.7)	Yes	50 (16.7)
3000–3999 RMB	87 (29.0)	No	170 (56.7)
4000–5000 RMB	164 (54.7)	Uncertain	80 (26.7)
> 5000 RMB	47 (15.7)	Type of certificate	
Daily working hours		No certificate	62 (20.7)
< 8 h	1 (0.3)	Junior	201 (67.0)
8–9 h	17 (5.7)	Intermediate	32 (10.7)
10–12 h	202 (67.3)	Advanced	5 (1.7)
> 12 h	80 (26.7)	Number of elderlies cared for	
Years of working in this occupation		1	6 (2.0)
< 1 Year	52 (17.3)	2–5	21 (7.0)
1–3 Years	103 (34.3)	5–8	222 (74.0)
3–5 Years	86 (28.7)	8–10	46 (15.3)
> 5 Years	59 (19.7)	> 10	5 (1.7)
Receive formal training			
Yes	282 (94.0)		
No	18 (6.0)		

*LTCF, long-term care facility

## Results

A total of 300 HCAs were surveyed on-site and all of them completed the scale, with the response rate being 100%. No data were missing and no corrections were made. Scores of the SSS-C ranged from 25 to 75, and the mean score was 59.56 (SD = 7.29) for the supervisors.

### Reliability

The 15-item SSS-C had satisfactory internal consistency (Cronbach’s α coefficient = 0.852), split half reliability (Spearman-Brown coefficient = 0.834) and test–retest reliability (Pearson correlation coefficient = 0.784) (see Table 2). Corrected item–total correlation and Cronbach’s α coefficient if item deleted for SSS-C are demonstrated in Table 3. The item-to-item correlations were positive, in the 0.083–0.541 range (Table 4).

**Table 2:** The results of reliability analysis

Reliability	Total (15 items)	Total (11 items)	Factor I	Factor II	Factor III
Cronbach’ ɑ	0.852	0.816	0.723	0.588	0.663
Split-half reliability	0.834	0.775	0.759	0.579	0.672
Test–retest reliability	0.784	0.740	0.714	0.660	0.651

**Table 3:** Corrected item–total correlation and Cronbach’s α coefficient if item deleted for SSS-C

Item	Mean	Standard deviation	Corrected Item–total correlation	Cronbach’s α if item deleted
1	4.03	0.715	0.518	0.842
2	3.82	0.873	0.473	0.844
3	3.79	1.033	0.394	0.850
4	4.14	0.786	0.488	0.843
5	3.98	0.782	0.435	0.845
6	3.84	0.913	0.529	0.840
7	3.92	0.818	0.408	0.847
8	3.87	0.947	0.454	0.845
9	4.10	0.729	0.490	0.843
10	3.86	0.977	0.463	0.845
11	4.15	0.723	0.525	0.841
12	4.06	0.736	0.493	0.843
13	4.26	0.821	0.432	0.846
14	3.85	0.992	0.631	0.834
15	3.87	0.852	0.587	0.837

**Table 4:** Item-to-item correlations for 15-item SSS-C

Item	1	2	3	4	5	6	7	8	9	10	11	12	13	14	15
1	1.000														
2	.357	1.000													
3	.230	.267	1.000												
4	.314	.330	.267	1.000											
5	.228	.334	.174	.276	1.000										
6	.310	.325	.337	.312	.245	1.000									
7	.250	.196	.186	.257	.207	.296	1.000								
8	.253	.183	.130	.223	.209	.308	.254	1.000							
9	.232	.238	.174	.232	.290	.336	.288	.353	1.000						
10	.293	.136	.263	.287	.277	.305	.242	.346	.287	1.000					
11	.315	.392	.203	.280	.277	.276	.190	.239	.365	.271	1.000				
12	.372	.231	.140	.291	.240	.235	.231	.276	.269	.287	.447	1.000			
13	.272	.298	.083	.268	.267	.200	.121	.190	.241	.140	.345	.321	1.000		
14	.384	.286	.342	.345	.277	.361	.278	.371	.275	.334	.349	.389	.541	1.000	
15	.363	.312	.377	.257	.253	.378	.331	.348	.392	.276	.330	.306	.267	.472	1.000

### Construct validity

Construct validity was examined by EFA, and the Kaiser–Meyer–Olkin (KMO) measure of sampling adequacy for this analysis was satisfactory (= 0.887), indicating that the sample size for the EFA was adequate. The result of the Bartlett’s test of sphericity was significant (*χ*^2^ = 1125.922, *P* < 0.001), demonstrating a sufficient correlation to perform factor analysis. Using the Kaiser criterion, three factors were initially identified, and total variance explained was 48.128%. However, since the communality of item1(My supervisor recognizes my ability to deliver quality care), 4(My supervisor tries to understand my point of view when I speak to them), 5(My supervisor tries to meet my needs in such ways as informing me of what is expected of me when working with my residents) and 7(My supervisor keeps me informed of any major changes in the work environment or organization) was less than 0.4, they should be deleted in theory [14]. After deletion of these four items and a second exploratory factor analysis, three factors could still be extracted, accounting for 55.654% of the total variance (KMO = 0.843, *χ*^2^ = 806.668, *P* < 0.001) (Table 5).

**Table 5:** Results of exploratory factor analysis

Items	Communality	Factors		
		Factor 1	Factor 2	Factor 3
13. My supervisor respects me as a person	0.623	0.789		
11. My supervisor encourages me even in difficult situations	0.488	0.644		
12. My supervisor makes a point of expressing appreciation when I do a good job	0.502	0.619		
14. My supervisor makes time to listen to me	0.525	0.565		
1.My supervisor recognizes my ability to deliver quality care	0.387	0.451		
5.My supervisor tries to meet my needs in such ways as informing me of what is expected of me when working with my residents	0.284	0.417		
8. I can rely on my supervisor to be open to any remarks I may make to him/her	0.567		0.726	
10. My supervisor strikes a balance between clients/ families’ concerns and mine	0.444		0.622	
9. My supervisor keeps me informed of any decisions that were made in regards to my residents	0.451		0.603	
7.My supervisor keeps me informed of any major changes in the work environment or organization	0.376		0.542	
15. My supervisor recognizes my strengths and areas for development	0.487		0.469	
3. My supervisor knows me well enough to know when I have concerns about resident care	0.655			0.797
2. My supervisor tries to meet my needs	0.565			0.553
6. I can rely on my supervisor when I ask for help, for example, if things are not going well between myself and my co-workers or between myself and residents and/or their families	0.488			0.542
4.My supervisor tries to understand my point of view when I speak to them	0.378			0.458

Mean item scores of the 15-item SSS ranged from 3.79 to 4.26 (SD = 0.715 to 1.033; Table 3). The four items under Factor I represented “the supervisors’ need to build connections with staff that involved respecting them as individuals” [9], therefore, this factor was labeled “building connections with staff”. Factor II including three items represented “the supervisors were available to staff to listen and respond to their concerns, and that they kept staff informed of what was new on the unit” [9], therefore, this factor was labeled “being dependable”. Factor III was labeled “being empathic” because the items loaded on this factor represented “the supervisors try to understand their point of view, recognize and accommodate expressed needs, recognize their abilities, and help them develop” [9]. Coefficient alpha reliabilities on the items comprising the three factors were: Factor I = 0.723, Factor II = 0.588 and Factor III = 0.663. The coefficient alpha for the 11-item SSS was 0.816.

### Discriminant validity

The discriminant validity of the SSS-C varied significantly between sites. For example, within Facility D, scores of the SSS were significantly higher than those for Facility C (*F* = 4.791, *p* < 0.005; see Table 6).

**Table 6:** Divergent construct validity across the long-term care facilities

Facility	Type of LTCF	Number of beds	Number of HCAs	Number of supervisors	Supportive supervisory scores (mean ± SD)
A	Private	582	80	42	58.71 ± 5.61
B	Private	230	24	7	60.42 ± 5.54
C	Public	600	65	23	57.22 ± 8.19
D	Public	664	131	52	61.08 ± 7.69
Total		2076	300	124	59.56 ± 7.29
ANOVA					*F* = 4.791
[*P* value]					[0.003]

LTCF, long-term care facility; HCA, health care aides

## Discussion

This was the first study to sinicize and validate a Chinese version of the SSS. The psychometrics results supported the utility of the SSS-C. It can be used as a reliable and valid instrument to determine the supervisory support of the team leader within LTC settings. It can be used in LTCFs across China, so that cross-cultural comparisons of influencing factors for registered nurses’ supportive supervision can be made possible, of which results can be popularized internationally to improve the quality of resident care. The EFA procedures carried out on the 15-item SSS-C in the present study were performed on an adequate sample of HCAs [15], and accepted criteria were used to determine the best factor solution. After consultation with experts on long-term care, it has been suggested that these four items need to be further modified in future research due to poor communality or new items could be added for a more complete measure of supervisory support in the Chinese context.

The results of the three-factor rotated solution were compatible with the three dimensions upon which the original SSS was initially based [9], however, they were discrepant with the original SSS which only has two dimensions. The three factors (building connections with staff, being dependable, and being empathic) were consistent with the theoretical underpinning of the SSS. At the core of effective supervision is a supervisor’s ability to develop and maintain positive working relationships with each HCA (what the dimension “building connections with staff” represents) [8], which can enhance the connection, cooperation and team work among the nurse supervisors and HCAs, and may significantly influence the HCA turnover and patient outcomes [16, 17]. Supportive supervision was defined as the extent to which the leader demonstrated empathy and reliability (also referred to as dependability) with staff [8]. The other two dimensions “being dependable” and “being empathic” were consistent with the above concepts. When HCAs can depend on their supervisors to achieve, relate to and enjoy their work, it will be easier for them to be committed to their work and become devoted caregivers [18]. Nurse supervisors in the LTCFs can help the staff counteract the negative effects of work-related pressure, perform their best over the long-term using Mindfulness, Hope and Compassion [19]. Therefore, “being dependable” and “being empathic” were also very important qualities of the supervisors in the LTCFs.

The discriminant validity of the SSS-C was also examined relative to construct validity, which showed that the SSS-C was able to differentiate supportive behaviors of supervisors between different LTCFs. No concurrent measure was conducted because there were no appropriate instruments.

The study has a few limitations. First, this study used a convenience sample of HCAs from the LTCFs in Suzhou. Second, no concurrent measure was conducted to analyze the construct validity. Third, in future research, new items could be added through expert consultation to form a more comprehensive measure of supervisory support in the Chinese context.

## Conclusions

The reliability and validity of the Chinese version of the SSS were acceptable. A strong three-factor solution was obtained, which was consistent with the three dimensions upon which the original SSS was initially based. At the core of supportive supervision is the supervisor’s ability to develop and maintain relationships with the HCAs. Through their dependability and empathy, these relationships can prosper. The SSS-C can be used as a reliable and valid tool to measure the level of supportive supervision in the LTCFs, which may influence retention of HCAs and quality of resident care.

## Data Availability

The authors have full control of all primary data and agree to allow the journal to review the data if requested.
